# Cardiometabolic Comorbidities of Lichen Planus—A Cross-Sectional Comparative Study

**DOI:** 10.3390/diagnostics15162039

**Published:** 2025-08-14

**Authors:** Mihaela Paula Toader, Oana Mihaela Condurache Hrițcu, Cristina Colac Boțoc, Antonia Elena Huțanu, Cătălina Anca Munteanu, Roxana Paraschiva Ciobanu, Ștefan Vasile Toader, Alin Gabriel Colac, Elena Porumb Andrese, Daciana Elena Brănișteanu

**Affiliations:** 1 Discipline of Oral Medicine, Oral Dermatology, Grigore T. Popa University of Medicine and Pharmacy, 16 Universitatii Street, 700115 Iasi, Romania or toaderpaula@gmail.com (M.P.T.); oana.condurache-hritcu@umfiasi.ro (O.M.C.H.); 2 Dermatology Clinic, University Clinical Railways Hospital, 1 Garabet Ibraileanu Street, 700115 Iasi, Romania; antoniaclivet@yahoo.com (A.E.H.); anca.munteanu2@yahoo.com (C.A.M.); r.p.ciobanu@gmail.com (R.P.C.); 3 Discipline of Physiopathology, Grigore T. Popa University of Medicine and Pharmacy, 16 Universitatii Street, 700115 Iasi, Romania; stefan.toader@umfiasi.ro; 4 Oral and Maxillo-Facial Surgery Clinic, “Sf. Spiridon” Emergency County Hospital, 700111 Iasi, Romania; colac.alin@yahoo.com; 5 Discipline of Dermatology, Grigore T. Popa University of Medicine and Pharmacy, 16 Universitatii Street, 700115 Iasi, Romania

**Keywords:** psoriasis, lichen planus, metabolic syndrome, cardiovascular disease

## Abstract

**Background/Objectives:** Cardiovascular disease (CVD) remains one of the leading causes of death worldwide, with several well-established risk factors. Among dermatological conditions, psoriasis is a well-known contributor to cardiometabolic risk, while lichen planus (LP) remains an underexplored chronic inflammatory disorder in this context. This study aimed to comparatively assess the prevalence and clinical patterns of metabolic syndrome (MetS) components in patients with LP versus psoriasis and healthy controls, focusing on the intrinsic inflammatory burden in patients not receiving systemic therapy. We also examined whether specific clinical subtypes of LP carry distinct metabolic profiles. **Methods:** We conducted a cross-sectional observational study at a tertiary dermatology center between January 2020 and December 2024. A total of 236 adult patients were included: 78 with LP, 79 with psoriasis, and 79 controls with minor dermatological conditions. Demographic, clinical, and laboratory data were collected. LP subtypes (cutaneous, mucocutaneous, reticular oral, erosive oral) were evaluated using the Lichen Planus Activity Index (LPAI) and Oral Lichen Planus Clinical Index (OLP-CI); psoriasis severity was assessed using the Psoriasis Area and Severity Index (PASI). Cardiometabolic comorbidities were assessed according to established guidelines. **Results:** LP patients showed significantly higher prevalence of hypertension (OR 1.94, *p* = 0.044) and type 2 diabetes mellitus (OR 3.09, *p* = 0.015) compared to controls. Compared to psoriasis, LP was associated with a higher prevalence of mixed dyslipidemia (OR 3.41, *p* = 0.033), while psoriasis showed more abdominal obesity (OR 0.35, *p* = 0.003). Mucosal LP subtypes, especially erosive and reticular oral LP, were linked to elevated cardiometabolic risk. **Conclusions:** LP, particularly its oral subtypes, is associated with a distinct cardiometabolic risk profile comparable to or exceeding that of psoriasis. These findings support the need for systematic metabolic screening in LP patients as part of comprehensive care.

## 1. Introduction

Cardiovascular disease (CVD) remains the leading cause of mortality in Europe, accounting for over 1.7 million deaths in 2021, approximately 32% of all deaths. Although age-standardized mortality rates from circulatory diseases have declined by around 20% across the European Union (EU) in the past decade, marked disparities persist between countries. Romania, for instance, continues to report one of the highest cardiovascular mortality rates in the EU—up to seven times greater than those observed in Western European countries such as France and Spain. Furthermore, Romania recorded only a 3% reduction in CVD mortality between 2011 and 2021, one of the lowest declines in the region. The country also exhibits high prevalence of modifiable cardiovascular risk factors, with 58% of men and 41% of women classified as overweight, among the highest rates in the EU, and it reports elevated short-term mortality following acute myocardial infarction. These findings reflect a substantial and persistent cardiovascular burden, particularly in socioeconomically disadvantaged populations [1]. This prompts to the necessity of periodic screening of the known cardiovascular risk factors, as well as the identification of emerging ones, in order to educate the population and to prevent major cardiovascular events leading to death.

Psoriasis is increasingly recognized as a systemic inflammatory disease rather than merely a cutaneous condition and considered an independent risk factor for metabolic syndrome and CVD. The so-called “skin march” describes the progression from localized psoriatic inflammation to systemic immune activation [2]. Key pro-inflammatory cytokines, such as tumor necrosis factor-alpha (TNF-α), interleukin (IL)-17, and IL-23, enter systemic circulation, promoting endothelial dysfunction, oxidative stress, insulin resistance, and a prothrombotic state. IL-17, for instance, binds to receptors on endothelial cells and upregulates chemokines and adhesion molecules, while TNF-α enhances IL-17 activity and disrupts insulin receptor signaling. Together, these mediators contribute to the development of cardiovascular and metabolic comorbidities, including atherosclerosis and hypertension. Consequently, routine metabolic screening has been integrated into clinical guidelines for patients with moderate-to-severe psoriasis [3,4,5,6].

A link between chronic inflammation and CVD is metabolic syndrome (MetS), a cluster of metabolic abnormalities defined by the presence of at least three of the following five components: abdominal obesity, elevated fasting glucose, high blood pressure, elevated triglycerides, and low levels of high-density lipoprotein (HDL) cholesterol. The National Cholesterol Education Program Adult Treatment Panel III (NCEP ATP III) and International Diabetes Federation (IDF) criteria provide widely accepted diagnostic frameworks [7,8].

In psoriasis, the IL-17/IL-23 axis, TNF-α, and interleukin-6 (IL-6) are known to contribute to the development of MetS [9]. Adipose tissue itself acts as a source of pro-inflammatory mediators, further sustaining systemic inflammation. Obesity, dyslipidemia, and insulin resistance are frequently observed in psoriatic patients and add significantly to their cardiovascular risk profile [10,11].

In contrast, lichen planus (LP) remains underexplored in this context, despite notable immunologic parallels with psoriasis. LP is a T helper type 1 (Th1)-dominant inflammatory disease, involving the activation of toll-like receptors, natural killer (NK) cells, dendritic cells, macrophages, and cytokines such as interferon-alpha (IFN-α), IL-17, IL-23, IL-6, and TNF-α. These shared inflammatory pathways suggest the potential for systemic metabolic involvement in LP as well. Preliminary evidence indicates increased glucose levels, dyslipidemia, and higher body mass index in patients with LP compared to healthy individuals [12,13,14]. Mucosal subtypes of LP (particularly erosive and reticular oral forms) may represent more systemically active variants, possibly associated with greater metabolic risk, although data remain limited [15,16].

This study aims to comparatively assess the prevalence and clinical patterns of metabolic syndrome components in patients with LP compared to psoriasis, and healthy controls. By focusing on individuals not currently receiving systemic therapies, we seek to isolate the intrinsic inflammatory burden of each condition and its relationship with cardiometabolic risk. We further explore whether specific clinical variants of lichen planus (cutaneous, mucocutaneous, or oral) carry differing metabolic profiles. Disease severity is assessed using the Psoriasis Area and Severity Index (PASI) for psoriasis, and the Lichen Planus Activity Index (LPAI) and Oral Lichen Planus Clinical Index (OLP-CI) for lichen planus. Through this analysis, we aim to identify potential contributors and risk factors to the development of cardiovascular disease and clarify whether metabolic screening protocols should be extended to LP as part of comprehensive patient care.

## 2. Materials and Methods

We conducted a cross-sectional, observational study at a single tertiary dermatology center between January 2020 and December 2024. During this period, 386 patients with psoriasis and 137 with LP were diagnosed, and 122 patients with minor dermatological diseases.

Inclusion criteria were as follows: age ≥18 years and histopathological confirmation of LP or psoriasis for the respective groups.

Exclusion criteria were as follows: lack of histopathological confirmation, concurrent autoimmune or chronic inflammatory conditions, primary genetic dyslipidemia, history of malignancy, chronic liver or kidney disease, active infection, pregnancy or lactation, and incomplete clinical or laboratory data. Patients receiving systemic therapy (e.g., immunosuppressants, biologics, corticosteroids) within the past 6 months were excluded to eliminate treatment-related confounding, as these agents can alter lipid and glucose metabolism, blood pressure, and systemic inflammation.

Of the eligible population, 157 participants were included: 78 patients with LP (group 1) and 79 patients with psoriasis (group 2). The control group (group 3) consisted of 79 patients with minor dermatologic conditions (benign tumors, acne, rosacea, tinea unguium) without systemic inflammatory disease. Details of the diagnostic groups included in the study are shown in Table 1.

**Figure 1:**
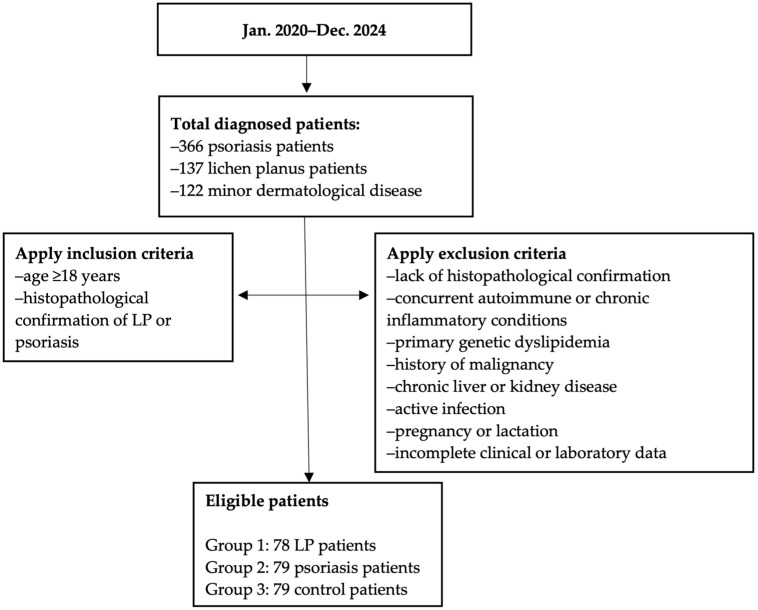
Flow diagram of the patient selections process, showing the screening, eligibility assessment and final inclusion steps.

The patient selection process is shown in Figure 1, outlining the sequential stages from initial screening to the final number of participants included in the study cohort.

Demographic data (age, sex) and anthropometric parameters (including waist circumference) were collected. Metabolic comorbidities were assessed through medical history, physical examination, and laboratory findings, and included arterial hypertension (HTN), type 2 diabetes mellitus (T2DM), hypercholesterolemia, mixed dyslipidemia, low high-density lipoprotein (HDL) cholesterol, hypertriglyceridemia, and abdominal obesity. Diagnostic definitions followed established guidelines (e.g., NCEP ATP III and IDF), based on clinical documentation or the use of chronic medication.

LP patients were subclassified into clinical subtypes: cutaneous LP, mucocutaneous LP, reticular oral LP, and erosive oral LP. Cutaneous LP severity was measured using the LPAI score, with scores 0–4 indicating mild, 5–8 moderate, and >9 severe disease. Oral LP was evaluated using the OLP-CI score, where scores of 0–2 were considered mild, 3–5 moderate, and >6 severe.

Psoriasis severity was assessed using PASI score, with scores < 7 classified as mild, 7–12 as moderate, and >12 as severe.

Descriptive statistics were used to summarize baseline characteristics. Categorical variables were compared using Pearson’s chi-squared test with Fisher correction if necessary. Odds ratios (ORs) and 95% confidence intervals (CIs) were calculated to estimate the association between disease status and metabolic parameters. Subgroup analyses were conducted for each LP subtype in comparison to both the psoriasis and control groups. Statistical significance was set at *p* < 0.05. Analyses were performed using SPSS Inc. Statistics for Windows, version 29.0 (Chicago, IL, USA).

## 3. Results

### 3.1. Demographic Characteristics of the Study Population by Diagnostic Group

The study included 236 patients divided into three cohorts: control (*n* = 79), LP (n = 78), and psoriasis (*n* = 79) (Table 2). All patients received topical treatment only; no systemic therapies were administered across groups.

Among the 236 participants, 96 (40.7%) were male and 140 (59.3%) were female. Sex distribution varied between groups (*p* < 0.001). The psoriasis group had the highest proportion of males (*n* = 49/79, 62.0%), while the LP group had the lowest (*n* = 20/78, 25.6%), with females representing the majority in both the control (*n* = 52/79, 65.8%) and lichen planus (*n* = 58/78, 74.4%) groups. In the LP group, over half of the patients (51.3%, *n* = 40/78) were older than 60, while in the psoriasis group, only 16.5% (*n* = 13/79) were in this age category. The psoriasis group had the highest proportion of participants aged 41–60 years (*n* = 40/79, 50.6%) and a relatively younger profile overall (*n* = 26/70, 32.9%), while the LP group had an older demographic. The control group showed a more balanced age distribution: 35.4% (*n* = 28/79) were under 40 years, 34.2% (*n* = 24/79) were between 41 and 60 years old, and 30.4% (*n* = 24/79) were over 40 years old (Table 3).

The mean age of the study population was 50.72 years (range: 18–86), with statistically significant differences observed among the groups (*p* < 0.001). Patients with LP had the highest mean age (57.54 years), while those with psoriasis were the youngest (mean age 46.00 years); control group participants had an intermediate mean age of 48.71 years (Table 4).

### 3.2. Clinical Characteristics of the Study Population by Diagnostic Group

Among patients with LP, 69.2% (*n* = 54/78) presented with the reticular oral subtype, 37.2% (*n* = 29/78) with the erosive oral form, 67.9% (*n* = 53/78) had cutaneous involvement, and 35.9% (*n* = 28/78) presented with a combined cutaneous–mucosal phenotype (Table 5).

Severity assessments revealed that mild disease was most frequent in oral LP (60.3%, *n* = 47/78), followed by cutaneous LP (32.1%, *n* = 25/78) and psoriasis (13.9%, *n* = 11/79). Moderate disease was found in 42.3% (*n* = 33/78) of cutaneous LP, 24.4% (*n* = 19/78) of oral LP, and 13.9% (*n* = 11/79) of psoriasis patients. Severe disease was found in 72.2% (*n* = 57/79) of psoriasis patients as per PASI scoring, compared to 25.6% (*n* = 20/78) of LP patients based on the LPAI and 15.4% (*n* = 12/78) of oral LP patients evaluated by the OLP-CI index (Table 6).

### 3.3. Distribution of Cardiometabolic Comorbidities Across Diagnostic Groups

HTN was the most common comorbidity, affecting 39.0% (*n* = 92/236) of the entire cohort. It was most frequent among LP patients (48.7%, *n* = 38/78), followed by those with psoriasis (35.4%, *n* = 28/79) and controls (32.9%, *n* = 26/79), though the intergroup difference did not reach statistical significance (*p* = 0.093).

T2DM was significantly more prevalent in the LP group (23.1%, *n* = 18/78) compared to the control group (8.9%, *n* = 7/79) and psoriasis group (15.2%, *n* = 12/79) (*p* = 0.049).

Hypercholesterolemia was identified in 24.6% of patients (*n* = 58/236), more commonly in the psoriasis (29.1%, *n* = 23/79) and LP groups (28.2%, *n* = 22/78) than in controls (16.5%, *n* = 12/79), without statistical significance (*p* = 0.120).

Mixed dyslipidemia was present in 10.6% (*n* = 25/236) of the total population, with highest frequency in LP (15.4%, *n* = 12/78), followed by control (11.4%, *n* = 9/79) and psoriasis (5.1%, *n* = 4/79), without statistical significance (*p* = 0.106). Low HDL levels were detected in 14.0% (*n* = 33/236) of patients, with similar rates in controls and psoriasis (16.5%, *n* = 13/78), and lower prevalence in LP (9.0%, *n* = 7/78) (*p* = 0.297). Hypertriglyceridemia was infrequent overall (4.2%, *n* = 10/236) and showed no significant group differences (*p* = 0.485).

Abdominal obesity was significantly more frequent in the psoriasis group (44.3%, *n* = 35/79) compared to LP (21.8%, *n* = 17/78) and controls (16.5%, *n* = 13/79) (*p* < 0.001).

Topical treatment was administered to 100% (*n* = 236/236) of patients across all groups (psoriasis, LP, and controls), while systemic treatment was uniformly absent (Table 7).

**Figure 2:**
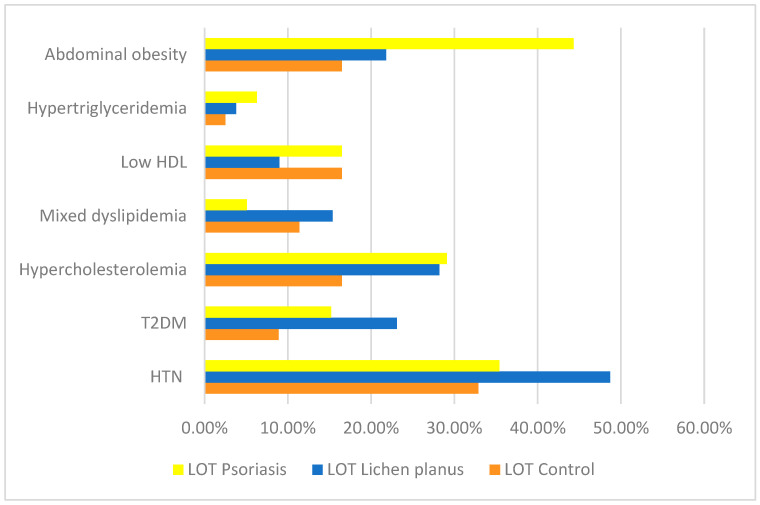
Cardiometabolic comorbidities across diagnostic groups.

When comparing patients with LP to healthy controls, statistically significant associations were observed for HTN and T2DM. HTN was present in 59.4% of LP patients (*n* = 38/64) compared to 40.6% of controls (*n* = 26/64), yielding an OR of 1.94 (95% CI: 1.015–3.695; *p* = 0.044), suggesting a nearly twofold increased likelihood of hypertension in the LP group. Similarly, T2DM was significantly more prevalent in LP patients (72.0%; *n* = 18/25) than in controls (28.0%; *n* = 7/25), with an OR of 3.09 (95% CI: 1.208–7.882; *p* = 0.015), indicating a threefold increased risk (Figure 2). Although hypercholesterolemia was more common in LP patients (62.9%; *n* = 22/35) than in controls (37.1%; *n* = 13/35), the difference did not reach statistical significance (*p* = 0.077). No significant differences were found between the LP and control groups in the prevalence of mixed dyslipidemia (57.1% vs. 42.9%; *p* = 0.462; *n* = 12/21 and *n* = 9/21, respectively), low HDL cholesterol (35.0% vs. 65.0%; *p* = 0.160; *n* = 7/20 and *n* = 13/20), hypertriglyceridemia (60.0% vs. 40.0%; *p* = 0.681; *n* = 3/5 and *n* = 2/5), or abdominal obesity (56.7% vs. 43.3%; *p* = 0.395; *n* = 17/30 and *n* = 13/30) (Figure 2) (see Appendix A, Table A1).

When comparing the LP group to the psoriasis group, statistically significant differences were observed in the prevalence of mixed dyslipidemia and abdominal obesity. Mixed dyslipidemia was more frequently identified in LP patients (75.0%; *n* = 12/16) compared to those with psoriasis (25.0%; *n* = 4/16), with a statistically significant association (*p* = 0.033) and an OR of 3.41 (95% CI: 1.049–11.083), indicating that LP patients were over three times more likely to present with mixed dyslipidemia. Conversely, abdominal obesity was significantly more prevalent in psoriasis patients (67.3%; *n* = 35/52) than in those with LP (32.7%; *n* = 17/52), with *p* = 0.003 and an OR of 0.35 (95% CI: 0.174–0.704), suggesting that LP patients were significantly less likely to exhibit abdominal obesity. No statistically significant differences were found between the groups regarding HTN (57.6% in LP vs. 42.4% in psoriasis; *p* = 0.092), T2DM (60.0% in LP vs. 40.0% in psoriasis; *p* = 0.209), hypercholesterolemia (48.9% in LP vs. 51.1% in psoriasis; *p* = 0.900), low HDL cholesterol (35.0% in LP vs. 65.0% in psoriasis; *p* = 0.160), or hypertriglyceridemia (37.5% in LP vs. 62.5% in psoriasis; *p* = 0.719) (Figure 2) (see Appendix A, Table A2).

### 3.4. Cardiometabolic Comorbidities According to LP Subtypes

**Figure 3:**
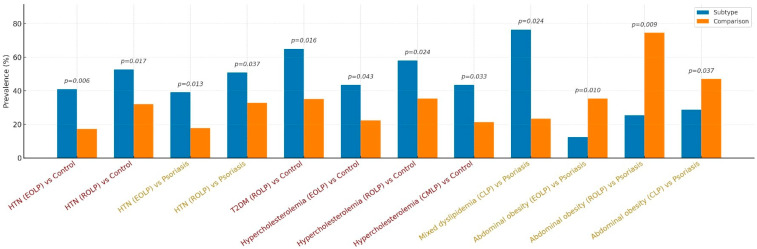
Prevalence of the MetS components in LP subtypes compared with controls and psoriasis patients.

HTN was significantly more frequent in patients with EOLP compared to controls (40.9%, *n* = 18/44 vs. 17.2%, *n* = 11/64; OR = 3.34, 95% CI: 1.38–8.08; *p* = 0.006) and also versus psoriasis (39.1%, *n* = 18/46 vs. 17.7%, *n* = 11/62; OR = 2.98, 95% CI: 1.24–7.19; *p* = 0.013). Similarly, ROLP was associated with a significantly higher prevalence of HTN compared to controls (52.7%, *n* = 29/55 vs. 32.1%, *n* = 25/78; OR = 2.37, 95% CI: 1.16–4.82; *p* = 0.017) and to psoriasis (50.9%, *n* = 29/57 vs. 32.9%, *n* = 25/76; OR = 2.11, 95% CI: 1.04–4.28; *p* = 0.037). No significant associations were identified for HTN in CLP or MCLP compared to either control or psoriasis groups (Figure 3).

For T2DM, ROLP patients showed a significantly increased risk compared to controls (65.0%, *n* = 13/20 vs. 35.0%, *n* = 7/20; OR = 3.26, 95% CI: 1.21–8.83; *p* = 0.016) (Figure 3), although comparisons with psoriasis (52.0%, *n* = 13/25 vs. 48.0%, *n* = 12/25; *p* = 0.198) did not reach statistical significance. No other LP subtypes showed significant differences in diabetes prevalence. 

Hypercholesterolemia was more prevalent in patients with EOLP than in controls (43.5%, *n* = 10/23 vs. 22.4%, *n* = 19/85; OR = 2.67, 95% CI: 1.01–7.05; *p* = 0.043). ROLP also demonstrated a higher risk (58.1%, *n* = 18/31 vs. 35.3%, *n* = 36/102; OR = 2.54, 95% CI: 1.12–5.77; *p* = 0.024), as did CMLP (43.5%, *n* = 10/23 vs. 21.4%, *n* = 18/84; OR = 2.82, 95% CI: 1.06–7.48; *p* = 0.033) (Figure 3). No statistically significant differences were observed in these subtypes compared to psoriasis.

A significant difference in mixed dyslipidemia was noted for CLP compared to psoriasis (69.2%, *n* = 9/13 vs. 30.8%, *n* = 4/13; OR = 3.84, 95% CI: 1.12–13.19; *p* = 0.024), suggesting that CLP patients were more likely to exhibit lipid derangements (Figure 3).

Abdominal obesity was significantly less prevalent in EOLP patients than in those with psoriasis (12.5%, *n* = 5/40 vs. 35.3%, *n* = 24/68; OR = 0.26, 95% CI: 0.09–0.76; *p* = 0.010). Similar trends were observed for ROLP (25.5%, *n* = 12/47 vs. 74.5%, *n* = 35/47; OR = 0.36, 95% CI: 0.17–0.78; *p* = 0.009) and CLP (28.6%, *n* = 14/49 vs. 47.0%, *n* = 35/83; OR = 0.45, 95% CI: 0.21–0.96; *p* = 0.037). No significant differences in abdominal obesity were found in comparisons between these subtypes and controls (see Appendix A, Table A3).

Compared to controls, HTN was more frequent in the ROLP group (52.7%, *n* = 29/55 vs. 32.1%, *n* = 25/78; OR = 2.37, 95% CI: 1.16–4.82; *p* = 0.017), as was T2DM (65.0%, *n* = 13/20 vs. 35.0%, *n* = 7/20; OR = 3.26, 95% CI: 1.21–8.83; *p* = 0.016), and hypercholesterolemia (58.1%, *n* = 18/31 vs. 35.3%, *n* = 36/102; OR = 2.54, 95% CI: 1.12–5.77; *p* = 0.024) (Figure 3).

In comparison with psoriasis, HTN remained significantly more prevalent in the ROLP group (50.9%, *n* = 29/57 vs. 32.9%, *n* = 25/76; OR = 2.11, 95% CI: 1.04–4.28; *p* = 0.037), while abdominal obesity was significantly less frequent in ROLP (25.5%, *n* = 12/47) compared to psoriasis (74.5%, *n* = 35/47; OR = 0.36, 95% CI: 0.17–0.78; *p* = 0.009), suggesting a lower risk of visceral adiposity in this subgroup (Figure 3).

No statistically significant differences were observed between ROLP and either control or psoriasis groups for the following parameters: mixed dyslipidemia (vs. control: 30.8%, *n* = 4/13 vs. 69.2%, *n* = 9/13, *p* = 0.447; vs. psoriasis: 50.0%, *n* = 4/8 vs. 50.0%, *n* = 4/8, *p* = 0.715), low HDL cholesterol (vs. control: 31.6%, *n* = 6/19 vs. 68.4%, *n* = 13/19, *p*= 0.387; vs. psoriasis: 31.6%, *n* = 6/19 vs. 68.4%, *n* = 13/19, *p* = 0.387), or hypertriglyceridemia (vs. control: 50.0%, *n* = 2/4 vs. 50.0%, *n* = 2/4, *p* = 1.000; vs. psoriasis: 28.6%, *n* = 2/7 vs. 71.4%, *n* = 5/7, *p* = 0.700) (see Appendix A, Table A4).

**Figure 4:**
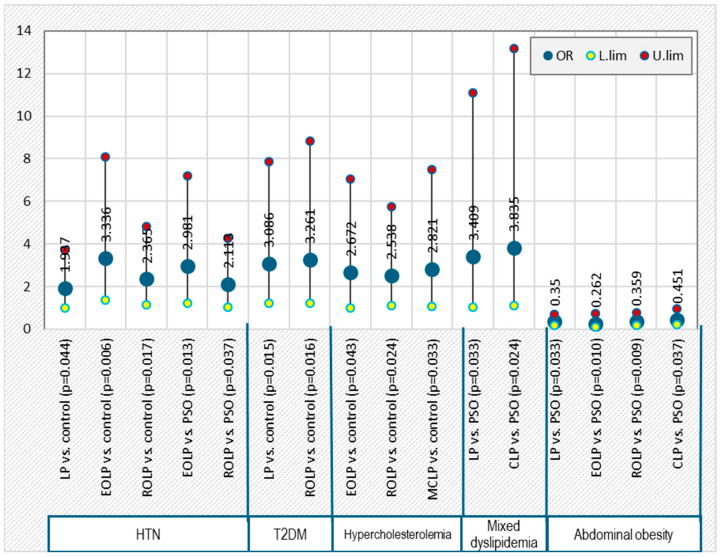
Significant risk of cardiometabolic comorbidities associated with the clinical subtypes of lichen planus compared with psoriasis and controls.

Patients with LP exhibited a higher risk of developing HTN (OR = 1.937, *p* = 0.044) and T2DM (OR = 3.086, *p* = 0.015) compared with controls and a higher risk of developing mixed dyslipidemia compared with psoriasis (OR = 3.409, *p* = 0.033) (Figure 4).

In patients with EOLP, there is an increased risk of HTN compared both with controls (OR = 3.336, *p* = 0.006) and with psoriasis (OR = 2.981, *p* = 0.013) and higher risk of hypercholesterolemia (OR = 2.672, *p* = 0.043) compared with controls (Figure 4).

ROLP was associated with a higher risk of HTN compared both with controls (OR = 2.365, *p* = 0.017) and psoriasis (OR = 2.113, *p* = 0.037), as well as a higher risk of T2DM (OR = 3.261, *p* = 0.016) and hypercholesterolemia (OR = 2.538, *p* = 0.024) compared with controls (Figure 4).

MCLP was associated with an increased risk of hypercholesterolemia compared with controls (OR = 2.821, *p* = 0.033) (Figure 4).

CLP patients showed a higher risk of mixed dyslipidemia compared with psoriasis (OR = 3.835, *p* = 0.024) (Figure 4).

### 3.5. Cardiometabolic Comorbidities According to Disease Severity

The prevalence of hypercholesterolemia was highest in patients with severe disease (45.5%, *n* = 10/22), followed by those with moderate (31.8%, *n* = 7/22) and mild LP (22.7%, *n* = 5/22). This trend suggests that higher cutaneous LP severity correlates with an increased likelihood of hypercholesterolemia. No statistically significant associations were found between LPAI severity scores and other metabolic parameters, including HTN (42.1%, *n* = 16/38 in mild vs. 21.1%, *n* = 8/38 in severe; *p* = 0.177), T2DM (44.4%, *n* = 8/18 in mild vs. 27.8%, *n* = 5/18 in severe; *p* = 0.312), mixed dyslipidemia (33.3%, *n* = 4/12 in mild vs. 16.7%, *n* = 2/12 in severe; *p* = 0.722), low HDL (42.9%, *n* = 3/7 in mild vs. 28.6%, *n* = 2/7 in severe; *p* = 0.723), hypertriglyceridemia (33.3%, *n* = 1/3 across all severity categories; *p* = 0.936), or abdominal obesity (23.5%, *n* = 4/17 in mild vs. 29.4%, *n* = 5/17 in severe; *p* = 0.695) (Table 8).

In patients with oral LP, assessed using the OLP-CI, no statistically significant associations were found between disease severity and any of the evaluated metabolic comorbidities. Specifically, the following *p*-values were observed: HTN (*p* = 0.407), T2DM (*p* = 0.657), hypercholesterolemia (*p* = 0.499), mixed dyslipidemia (*p* = 0.626), low HDL (*p* = 0.548), hypertriglyceridemia (*p* = 0.357), and abdominal obesity (*p* = 0.810).

Among patients with HTN, 75.0% (*n* = 21/28) had severe PASI scores, compared to 70.6% (*n* = 36/51) of those without hypertension (*p* = 0.375). For T2DM, 75.0% (*n* = 9/12) of affected individuals had severe disease, versus 71.6% (*n* = 48/67) of those without diabetes (*p* = 0.815). Similarly, hypercholesterolemia was present in 78.3% (*n* = 18/23) of severe cases, compared to 69.6% (*n* = 39/56) in the non-hypercholesterolemia group (*p* = 0.663). For other metabolic parameters, the pattern remained consistent but nonsignificant: mixed dyslipidemia was observed in 75.0% (*n* = 3/4) of severe psoriasis cases (*p* = 0.618), low HDL cholesterol in 61.5% (*n* = 8/13; *p* = 0.548), hypertriglyceridemia in 100% (*n* = 5/5; *p* = 0.357), and abdominal obesity in 77.1% (*n* = 27/35; *p* = 0.469). Across all these comparisons, the distribution of mild and moderate severity scores was lower and did not significantly differentiate the presence or absence of metabolic conditions (Table 9).

## 4. Discussion

This study provides a comparative analysis of the prevalence and clinical characteristics of MetS in patients with psoriasis and LP who were not treated with systemic targeted therapies, as well as in healthy controls. Chronic low-grade systemic inflammation plays a pivotal role in the pathogenesis of MetS. Adipose tissue, especially in the context of obesity, acts as an immunologically active organ, releasing pro-inflammatory cytokines such as TNF-α, IL-6, and IL-1β, which impair insulin signaling, promote endothelial dysfunction, and contribute to vascular inflammation [17,18]. These processes are mirrored in patients with chronic inflammatory skin diseases, notably psoriasis and LP, both of which are increasingly recognized as systemic immune disorders rather than isolated dermatologic conditions.

This inflammatory state, often termed “metaflammation,” is hypothesized to underlie the development of metabolic complications in individuals with chronic immune-mediated diseases. Importantly, systemic inflammation may not only be a consequence but also a precursor to metabolic derangements. Elevated levels of CRP and IL-6 have been found to precede the onset of T2DM and CVD, suggesting that inflammatory dermatoses like psoriasis and LP may serve as early markers for cardiometabolic screening and intervention [19,20].

In our study, a statistically significant association was found between LP and both HTN (OR = 1.94, 95% CI: 1.015–3.695, *p* = 0.044) and T2DM (OR = 3.09, 95% CI: 1.208–7.882, *p* = 0.015), when compared to healthy controls. Additionally, mixed dyslipidemia was more prevalent in LP than in psoriasis (OR = 3.41, 95% CI: 1.049–11.083, *p* = 0.033), while abdominal obesity was significantly more frequent in the psoriasis group compared to LP (OR = 0.35, 95% CI: 0.174–0.704, *p* = 0.003). These findings underscore the metabolic burden shared by both conditions, albeit with distinct profiles. Although psoriasis is traditionally viewed as the prototypical inflammatory dermatosis associated with metabolic syndrome, our findings suggest that LP, particularly mucosal subtypes, may impose a comparable or even higher burden of certain metabolic comorbidities. It is well established that moderate-to-severe psoriasis carries a higher risk of metabolic syndrome, largely due to its associated systemic inflammation and immune activation. This relationship may help explain the metabolic profile observed in our psoriasis cohort and supports the growing consensus that disease severity is an important modifier of cardiometabolic risk in psoriatic patients. While psoriasis was more strongly associated with abdominal obesity, LP patients demonstrated a higher prevalence of mixed dyslipidemia, hypercholesterolemia, and diabetes, particularly in the absence of systemic therapy. These differing profiles highlight the need for disease-specific screening strategies.

Emerging evidence suggests that up to 59% of patients with LP may develop MetS or its components, a notion supported by our findings [15]. The LP group had a higher prevalence of T2DM (23.1%) and hypercholesterolemia (28.2%) than controls, though the latter did not reach statistical significance. Furthermore, subgroup analysis revealed that specific clinical forms of LP, particularly the erosive and reticular oral variants, are associated with a significantly elevated risk of metabolic comorbidities. EOLP was associated with increased odds of HTN compared to both controls (OR = 3.34, *p* = 0.006) and psoriasis (OR = 2.98, *p* = 0.013), and also showed a higher likelihood of hypercholesterolemia (OR = 2.67, *p* = 0.043). Reticular oral LP was likewise linked to elevated risks for HTN (vs. control: OR = 2.37, *p* = 0.017; vs. psoriasis: OR = 2.11, *p* = 0.037), T2DM (OR = 3.26, *p* = 0.016), and hypercholesterolemia (OR = 2.54, *p* = 0.024). 

While LP is sometimes regarded as a disease with a shorter and potentially self-limited course compared to psoriasis, this generalization does not apply to all clinical forms. In particular, mucosal subtypes such as erosive oral LP, vulvovaginal LP, and sclero-atrophic variants are frequently chronic, relapsing, and resistant to standard treatment. These forms may persist for years, contributing to sustained systemic inflammation and long-term immune activation. Recent studies, along with our own findings, suggest that the development of MetS in LP may be driven not only by disease duration, but also by autoimmune mechanisms and mucosal involvement. Notably, mucosal LP, especially in its erosive and reticular forms, was associated with a higher prevalence of MetS components, indicating a more systemically active immunologic phenotype that may confer increased cardiometabolic risk [21].

Although LP severity is not routinely quantified using standardized scoring systems, our study evaluated metabolic comorbidities in relation to LP severity using LPAI and OLP-CI scores. Hypercholesterolemia was significantly more frequent in patients with severe LPAI scores (*p* = 0.042), supporting the concept that metabolic risk increases with disease burden. Other comorbidities, including HTN, T2DM, and dyslipidemia subtypes, did not show significant associations with LP severity. Similarly, OLP-CI severity scores showed no significant correlation with metabolic parameters, although trends were consistent with those observed in LPAI.

In psoriasis patients, while the disease is a well-known systemic inflammatory disorder, no statistically significant associations were observed between PASI severity and the prevalence of metabolic comorbidities. This suggests that metabolic risk may be more evenly distributed regardless of disease activity level, possibly due to the chronic and persistent nature of systemic inflammation in psoriasis.

Gender-specific differences have also been observed in the literature, with some studies reporting a higher frequency of MetS among female LP patients, and male predominance in psoriasis-associated metabolic risk [22,23]. In our cohort, psoriasis was indeed more common in males (62%), whereas LP and control groups were predominantly female, aligning with existing epidemiologic patterns and potentially influencing comorbidity distributions. These sex-specific patterns may carry important cardiovascular implications. Chronic inflammatory diseases such as LP, which disproportionately affect women, have been increasingly recognized as contributors to elevated cardiovascular risk. Persistent systemic inflammation in women has been linked to endothelial dysfunction, microvascular impairment, and heightened susceptibility to stress-induced vasospasm, all established mechanisms of myocardial ischemia and type 2 myocardial infarction. The loss of estrogen’s vasoprotective effects after menopause further amplifies this risk by reducing nitric oxide availability and enhancing pro-inflammatory cytokine expression, including IL-6 and TNF-α. Additionally, spontaneous coronary artery dissection, a leading cause of myocardial infarction in younger and peripartum women, has been associated with chronic immune-mediated diseases [24]. These mechanisms underscore the need for cardiovascular screening approaches tailored to women with chronic inflammatory dermatoses, particularly those with mucosal LP subtypes, where systemic inflammatory activity may be more pronounced.

In terms of lipid metabolism, LP has been linked to dysregulation, consistent with our findings [25]. Hypercholesterolemia and mixed dyslipidemia were notably prevalent in LP patients, with the latter showing a significantly increased odds compared to psoriasis. Studies by Kuntoji et al. and Shingla et al. have similarly reported elevated total cholesterol, triglycerides, and reduced HDL levels in LP patients, supporting the concept of systemic lipid imbalance as a key metabolic signature in this population [26,27].

HTN in LP has also been attributed to reduced prostacyclin synthesis, contributing to vascular dysfunction and elevated cardiovascular risk [28]. Although we did not directly measure prostacyclin levels, the association between LP and hypertension in our study supports this hypothesis.

The relationship between LP and hyperglycemia or T2DM remains controversial. While some studies have failed to find a significant association, our results indicated a higher prevalence of diabetes among LP patients compared to controls, particularly among those with reticular oral involvement, reinforcing the need for further investigation into this connection [29,30]. 

From an immunologic standpoint, both LP and psoriasis are Th1- and Th17-driven diseases, characterized by elevated IFN-γ, TNF-α, and IL-17 activity. These cytokines not only mediate local skin inflammation but also contribute to systemic metabolic disruption by impairing insulin signaling, increasing hepatic gluconeogenesis, and altering lipid profiles. The fact that mucosal LP subtypes showed stronger metabolic associations in our study may reflect a more intense or disseminated immune activation, potentially linked to systemic T-cell responses or plasmacytoid dendritic cell infiltration, mechanisms increasingly implicated in LP pathogenesis [29,31].

The comparative nature of this study offers valuable insights into how different chronic inflammatory dermatoses contribute to metabolic risk through both overlapping and distinct mechanisms. While the link between psoriasis and MetS is well established, our findings support growing evidence that LP, especially in its chronic, mucosal, or severe forms, may also impose a significant systemic burden.

Despite increasing evidence of systemic involvement, LP remains underrecognized in clinical practice as a condition warranting metabolic screening, leading to potential missed opportunities for early intervention and risk reduction. Dermatologists should consider routine screening for metabolic syndrome in patients with both psoriasis and LP, particularly those with mucosal involvement, obesity, or female sex. A minimum workup should include blood pressure measurement, waist circumference, fasting glucose, and lipid panel assessment. Early identification and management of metabolic abnormalities could mitigate long-term cardiovascular morbidity. These results underscore the importance of interdisciplinary collaboration between dermatologists, endocrinologists, and cardiologists.

Our results align with growing international consensus recommending cardiovascular and metabolic risk assessment in patients with chronic inflammatory dermatoses. The European Academy of Dermatology and Venereology (EADV) and American Academy of Dermatology (AAD) have both emphasized the need for proactive comorbidity screening in psoriasis; our findings suggest that a similar approach may be warranted for LP [32,33].

To our knowledge, this is one of the first studies to provide a comprehensive comparison of metabolic syndrome components across both psoriasis and multiple clinical subtypes of LP, using validated severity indices. By including erosive and reticular oral LP, cutaneous LP, and mucocutaneous forms, and stratifying by disease activity scores such as PASI, LPAI, and OLP-CI, this study offers a granular analysis of the systemic metabolic burden associated with LP. Furthermore, the exclusion of patients receiving systemic therapy allowed for an unconfounded assessment of intrinsic disease-related risk, strengthening the validity of our findings. These contributions expand current understanding of LP as a potentially systemic condition and support its inclusion in metabolic risk screening strategies alongside psoriasis.

This study has several notable strengths. It included a well-defined, treatment-naïve cohort across three balanced groups, psoriasis, LP, and healthy controls, eliminating confounding from systemic anti-inflammatory therapies. The stratification of LP by clinical subtype (erosive oral, reticular oral, cutaneous, and mucocutaneous) allowed for a nuanced assessment of metabolic comorbidity patterns. Moreover, the use of validated severity indices (PASI, LPAI, and OLP-CI) enabled standardized evaluation of disease burden across groups.

This study has several important limitations that should be acknowledged. First, its cross-sectional design precludes any causal inferences regarding the relationship between chronic inflammatory dermatoses and MetS components, and it does not allow assessment of temporal relationships between disease onset, severity, and metabolic outcomes. Longitudinal studies are needed to clarify whether cutaneous disease activity precedes or follows metabolic alterations. Second, lifestyle and behavioral factors, such as smoking, alcohol consumption, diet, physical activity, and socioeconomic status, were not recorded, despite their potential impact on metabolic risk profiles. Likewise, systemic inflammatory biomarkers (e.g., C-reactive protein, interleukin-6, tumor necrosis factor-α, adipokines) were not measured, limiting mechanistic insights into the link between systemic inflammation and metabolic dysfunction. Third, while patients receiving systemic dermatologic therapy were excluded, individuals undergoing treatment for metabolic conditions (e.g., antihypertensives, antidiabetics, statins) were included, which may have influenced lipid and glucose measurements. Fourth, the selection of the control group, patients with minor dermatologic conditions not currently associated with systemic inflammation, may have introduced residual confounding. The control group was not matched for comorbidities, lifestyle factors, age, or sex, and demographic imbalances were also present between groups. Notably, the LP cohort had the oldest mean age, and there was a higher proportion of female participants in the LP and control groups reflecting the demographic profile of patients admitted to our clinic during the study period. We acknowledge that significant age differences between the study groups may have influenced the prevalence of metabolic comorbidities, particularly in the LP group. Although this study was exploratory and descriptive, and age adjustment was not performed, this remains a potential confounder and should be taken into account in future multivariate analyses. The study did not include multivariate adjustments for age or sex, which may have influenced the observed associations between dermatologic diagnoses and metabolic comorbidities. We relied on bivariate analyses (Chi-square and crude ORs) due to the exploratory nature of the study and the limited sample size, which restricted the use of logistic regression models. The lack of multivariate adjustment represents a limitation and should be addressed in future studies on larger cohorts, where adjusted comparisons by demographic confounders can be performed for improved control.

Future research should prioritize prospective, multicenter, longitudinal studies with larger and demographically matched cohorts to clarify the temporal relationship between chronic dermatoses and metabolic disease progression. The development and validation of standardized disease severity indices for LP, particularly for oral and mucocutaneous subtypes, would enhance clinical monitoring and research consistency. Biomarker-based investigations, including IL-6, C-reactive protein, TNF-α, and adipokines, may provide mechanistic insights into the interplay between cutaneous inflammation and systemic metabolic dysfunction, as well as identify shared inflammatory pathways between dermatologic and metabolic diseases. Interventional trials evaluating whether systemic anti-inflammatory therapies can reverse or mitigate metabolic risk in LP and psoriasis are a high priority. Finally, given the distinct metabolic profiles observed between LP subtypes and psoriasis, tailored screening and management strategies should be considered in future clinical guidelines.

## 5. Conclusions

This study highlights the significant association between chronic inflammatory dermatoses, particularly psoriasis and LP, and components of metabolic syndrome. Notably, patients with LP, especially those with mucosal involvement such as erosive and reticular oral subtypes, demonstrated an increased prevalence of HTN, T2DM, hypercholesterolemia, and mixed dyslipidemia. These findings challenge the traditional perception of LP as a primarily localized condition and underscore its potential systemic impact. While psoriasis remains a well-established model for immune–metabolic interactions, our results suggest that LP may carry an equal or even greater metabolic burden in specific clinical contexts. These insights support the implementation of routine metabolic screening in LP, alongside established guidelines for psoriasis, and call for interdisciplinary management strategies. Future prospective and mechanistic studies are warranted to clarify causality, assess the impact of systemic treatments, and better define the inflammatory–metabolic interface in LP.

## Figures and Tables

**Table 1:** Distribution of patients across study groups and control subgroups.

Study Groups	Disease	No. Patients
**Group 1**	Lichen planus	78
**Group 2**	Psoriasis	79
**Group 3**	Benign tumors	44
	Acne	7
	Rosacea	16
	Tinea unguium	12

**Table 2:** Distribution of study participants by diagnostic group.

Lot	N	%
Control	79	33.5
Lichen planus	78	33.1
Psoriasis	79	33.5
Total	236	100.0

**Table 3:** Demographic characteristics of the study population by diagnostic group.

		Total		Lot						Pearson Chi-Squared Test *p*-Value
				Control		Lichen Planus		Psoriasis		
		N	%	N	%	N	%	N	%	
Gender	Male	96	40.7%	27	34.2%	20	25.6%	49	62.0%	<0.001
	Female	140	59.3%	52	65.8%	58	74.4%	30	38.0%	
Age groups	Under 40 years	64	27.1%	28	35.4%	10	12.8%	26	32.9%	<0.001
	41–60 years	95	40.3%	27	34.2%	28	35.9%	40	50.6%	
	Over 60 years	77	32.6%	24	30.4%	40	51.3%	13	16.5%	
Total		236	100.0%	79	100.0%	78	100.0%	79	100.0%	

**Table 4:** Descriptive statistics of age by diagnostic group.

	Lot	N	Mean	Standard Deviation	Minimum	Maximum	Median
Age	Control	79	48.71	16.613	19	75	52.00
	Lichen planus	78	57.54	14.148	21	86	61.00
	Psoriasis	79	46.00	14.314	18	73	47.00
	Total	236	50.72	15.795	18	86	53.00

**Table 5:** Distribution of clinical subtypes in LP group.

Clinical Subtypes of LP		N	%
**EOLP**	Present	29	37.2
	Absent	49	62.8
**ROLP**	Present	54	69.2
	Absent	24	30.8
**CLP**	Present	53	67.9
	Absent	25	32.1
**MCLP**	Present	28	35.9
	Absent	50	64.1
Total		78	100.0

EOLP: erosive oral lichen planus; ROLP: reticular oral lichen planus; CLP: cutaneous lichen planus; MCLP: muco-cutaneous lichen planus.

**Table 6:** Distribution of disease severity according to LPAI, OLP-CI, and PASI scores.

	Severity Score LPAI		Severity Score OLP-CI		Severity Score PASI	
	N	%	N	%	N	%
Mild	25	32.1	47	60.3	11	13.9
Moderate	33	42.3	19	24.4	11	13.9
Severe	20	25.6	12	15.4	57	72.2
Total	78	100.0	78	100.0	79	100.0

LPAI: lichen planus activity index; OLP-CI: oral lichen planus clinical index; PASI: psoriasis area severity index.

**Table 7:** Distribution of cardiometabolic comorbidities across diagnostic groups and Pearson Chi-Squared test results.

		Total		Lot						Pearson Chi-Squared Test *p*-Value
				Control		Lichen Planus		Psoriasis		
		N	%	N	%	N	%	N	%	
HTN	Present	92	39.0%	26	32.9%	38	48.7%	28	35.4%	0.093
	Absent	144	61.0%	53	67.1%	40	51.3%	51	64.6%	
T2DM	Present	37	15.7%	7	8.9%	18	23.1%	12	15.2%	0.049 *
	Absent	199	84.3%	72	91.1%	60	76.9%	67	84.8%	
Hypercholesterolemia	Present	58	24.6%	13	16.5%	22	28.2%	23	29.1%	0.120
	Absent	178	75.4%	66	83.5%	56	71.8%	56	70.9%	
Mixed dyslipidemia	Present	25	10.6%	9	11.4%	12	15.4%	4	5.1%	0.106
	Absent	211	89.4%	70	88.6%	66	84.6%	75	94.9%	
Low HDL	Present	33	14.0%	13	16.5%	7	9.0%	13	16.5%	0.297
	Absent	203	86.0%	66	83.5%	71	91.0%	66	83.5%	
Hypertriglyceridemia	Present	10	4.2%	2	2.5%	3	3.8%	5	6.3%	0.485
	Absent	226	95.8%	77	97.5%	75	96.2%	74	93.7%	
Abdominal obesity	Present	65	27.5%	13	16.5%	17	21.8%	35	44.3%	<0.001 **
	Absent	171	72.5%	66	83.5%	61	78.2%	44	55.7%	
Topical treatment	Present	236	100.0%	79	100.0%	78	100.0%	79	100.0%	
Systemic treatment	Absent	236	100.0%	79	100.0%	78	100.0%	79	100.0%	
Total		236	100.0%	79	100.0%	78	100.0%	79	100.0%	

HTN: arterial hypertension; T2DM: type 2 diabetes mellitus; HDL: high-density lipoprotein; *: statistically significant; **: highly statistically significant.

**Table 8:** Association between LPAI-based severity of cutaneous lichen planus and cardiometabolic comorbidities.

		HTN				Pearson Chi-Squared Test *p*-Value
		Present		Absent		
		N	%	N	%	
LPAI	Mild	16	42.1%	9	22.5%	0.177
	Moderate	14	36.8%	19	47.5%	
	Severe	8	21.1%	12	30.0%	
Total		38	100.0%	40	100.0%	
		**T2DM**				**Pearson Chi-squared test** ***p*** **-value**
		**present**		**absent**		
		**N**	**%**	**N**	**%**	
LPAI	Mild	8	44.4%	17	28.3%	0.312
	Moderate	5	27.8%	28	46.7%	
	Severe	5	27.8%	15	25.0%	
Total		18	100.0%	60	100.0%	
		**Hypercholesterolemia**				**Pearson Chi-squared test** ***p*** **-value**
		**present**		**absent**		
		**N**	**%**	**N**	**%**	
LPAI	Mild	5	22.7%	20	35.7%	0.042 *
	Moderate	7	31.8%	26	46.4%	
	Severe	10	45.5%	10	17.9%	
Total		22	100.0%	56	100.0%	
		**Mixed dyslipidemia**				**Pearson Chi-squared test** ***p*** **-value**
		**present**		**absent**		
		**N**	**%**	**N**	**%**	
LPAI	Mild	4	33.3%	21	31.8%	0.722
	Moderate	6	50.0%	27	40.9%	
	Severe	2	16.7%	18	27.3%	
Total		12	100.0%	66	100.0%	
		**Low HDL**				**Pearson Chi-squared test** ***p*** **-value**
		**present**		**absent**		
		**N**	**%**	**N**	**%**	
LPAI	Mild	3	42.9%	22	31.0%	0.723
	Moderate	2	28.6%	31	43.7%	
	Severe	2	28.6%	18	25.4%	
Total		7	100.0%	71	100.0%	
		**Hypertriglyceridemia**				**Pearson Chi-squared test** ***p*** **-value**
		**present**		**absent**		
		**N**	**%**	**N**	**%**	
LPAI	Mild	1	33.3%	24	32.0%	0.936
	Moderate	1	33.3%	32	42.7%	
	Severe	1	33.3%	19	25.3%	
Total		3	100.0%	75	100.0%	
		**Abdominal obesity**				**Pearson Chi-squared test** ***p*** **-value**
		**present**		**absent**		
		**N**	**%**	**N**	**%**	
LPAI	Mild	4	23.5%	21	34.4%	0.695
	Moderate	8	47.1%	25	41.0%	
	Severe	5	29.4%	15	24.6%	
Total		17	100.0%	61	100.0%	

LPAI: lichen planus activity index; HTN: hypertension; T2DM: type 2 diabetes mellitus; HDL: high-density lipoprotein.

**Table 9:** Correlation between PASI-based psoriasis severity and prevalence of metabolic syndrome components.

		HTN				Pearson Chi-Squared Test *p*-Value
		Present		Absent		
		N	%	N	%	
PASI	Mild	2	7.1%	9	17.6%	0.375
	Moderate	5	17.9%	6	11.8%	
	Severe	21	75.0%	36	70.6%	
Total		28	100.0%	51	100.0%	
		**T2DM**				**Pearson Chi-squared test** ***p*** **-value**
		**present**		**absent**		
		**N**	**%**	**N**	**%**	
PASI	Mild	1	8.3%	10	14.9%	0.815
	Moderate	2	16.7%	9	13.4%	
	Severe	9	75.0%	48	71.6%	
Total		12	100.0%	67	100.0%	
		**Hypercholesterolemia**				**Pearson Chi-squared test** ***p*** **-value**
		**present**		**absent**		
		**N**	**%**	**N**	**%**	
PASI	Mild	3	13.0%	8	14.3%	0.663
	Moderate	2	8.7%	9	16.1%	
	Severe	18	78.3%	39	69.6%	
Total		23	100.0%	56	100.0%	
		**Mixed dyslipidemia**				**Pearson Chi-squared test** ***p*** **-value**
		**present**		**absent**		
		**N**	**%**	**N**	**%**	
PASI	Mild	1	25.0%	10	13.3%	0.618
	Moderate	0	0.0%	11	14.7%	
	Severe	3	75.0%	54	72.0%	
Total		4	100.0%	75	100.0%	
		**Low HDL**				**Pearson Chi-squared test** ***p*** **-value**
		**present**		**absent**		
		**N**	**%**	**N**	**%**	
PASI	Mild	3	23.1%	8	12.1%	0.548
	Moderate	2	15.4%	9	13.6%	
	Severe	8	61.5%	49	74.2%	
Total		13	100.0%	66	100.0%	
		**Hypertriglyceridemia**				**Pearson Chi-squared test** ***p*** **-value**
		**present**		**absent**		
		**N**	**%**	**N**	**%**	
PASI	Mild	0	0.0%	11	14.9%	0.357
	Moderate	0	0.0%	11	14.9%	
	severe	5	100.0%	52	70.3%	
Total		5	100.0%	74	100.0%	
		**Abdominal obesity**				**Pearson Chi-squared test** ***p*** **-value**
		**present**		**absent**		
		**N**	**%**	**N**	**%**	
PASI	Mild	5	14.3%	6	13.6%	0.469
	Moderate	3	8.6%	8	18.2%	
	Severe	27	77.1%	30	68.2%	
Total		35	100.0%	44	100.0%	

PASI: psoriasis activity severity index; HTN: hypertension; T2DM: type 2 diabetes mellitus; HDL: high-density lipoprotein.

## Data Availability

The original contributions presented in this study are included in the article. Further inquiries can be directed to the corresponding authors.

## Abbreviations

The following abbreviations are used in this manuscript:

CVD	Cardiovascular Disease
EU	European Union
TNF-α	Tumor Necrosis Factor-alpha
IL	Interleukin
IL-6	Interleukin-6
IL-17	Interleukin-17
IL-23	Interleukin-23
Th1	T Helper Type 1
NK cells	Natural Killer Cells
IFN-α	Interferon-alpha
MetS	Metabolic Syndrome
NCEP ATP III	National Cholesterol Education Program Adult Treatment
Panel III	
IDF	International Diabetes Federation
HTN	Arterial Hypertension
T2DM	Type 2 Diabetes Mellitus
HDL	High-Density Lipoprotein
LP	Lichen Planus
PASI	Psoriasis Area and Severity Index
LPAI	Lichen Planus Activity Index
OLP-CI	Oral Lichen Planus Clinical Index
EOLP	Erosive Oral Lichen Planus
ROLP	Reticular Oral Lichen Planus
CLP	Cutaneous Lichen Planus
MCLP	Mucocutaneous Lichen Planus
OR	Odds ratio
CI	Confidence Interval
SPSS	Statistical Package for the Social Sciences

## Appendix A

**Table A1:** Comparison of cardiometabolic comorbidities between lichen planus and control groups with odds ratios and 95% confidence intervals.

		HTN				Pearson Chi-Squared Test *p*-Value	OR 95% CI
		Present		Absent			
		N	%	N	%		
**Group**	Lichen planus	38	59.4%	40	43.0%	0.044 *	1.937
	Control	26	40.6%	53	57.0%		1.015 ÷ 3.695
**Total**		64	100.0%	93	100.0%		
		**T2DM**				**Pearson Chi-squared test** ***p*** **-value**	**OR** **95% CI**
		**present**		**absent**			
		**N**	**%**	**N**	**%**		
**Group**	Lichen planus	18	72.0%	60	45.5%	0.015 *	3.086
	Control	7	28.0%	72	54.5%		1.208 ÷ 7.882
**Total**		25	100.0%	132	100.0%		
		**Hypercholesterolemia**				**Pearson Chi-squared test** ***p*** **-value**	**OR** **95% CI**
		**present**		**absent**			
		**N**	**%**	**N**	**%**		
**Group**	Lichen planus	22	62.9%	56	45.9%	0.077	-
	Control	13	37.1%	66	54.1%		
**Total**		35	100.0%	122	100.0%		
		**Mixed dyslipidemia**				**Pearson Chi-squared test** ***p*** **-value**	**OR** **95% CI**
		**present**		**absent**			
		**N**	**%**	**N**	**%**		
**Group**	Lichen planus	12	57.1%	66	48.5%	0.462	-
	Control	9	42.9%	70	51.5%		
**Total**		21	100.0%	136	100.0%		
		**Low HDL**				**Pearson Chi-squared test** ***p*** **-value**	**OR** **95% CI**
		**present**		**absent**			
		**N**	**%**	**N**	**%**		
**Group**	Lichen planus	7	35.0%	71	51.8%	0.160	-
	Control	13	65.0%	66	48.2%		
**Total**		20	100.0%	137	100.0%		
		**Hypertriglyceridemia**				**Pearson Chi-squared test** ***p*** **-value**	**OR** **95% CI**
		**present**		**absent**			
		**N**	**%**	**N**	**%**		
**Group**	Lichen planus	3	60.0%	75	49.3%	0.681	-
	Control	2	40.0%	77	50.7%		
**Total**		5	100.0%	152	100.0%		
		**Abdominal obesity**				**Pearson Chi-squared test** ***p*** **-value**	**OR** **95% CI**
		**present**		**absent**			
		**N**	**%**	**N**	**%**		
**Group**	Lichen planus	17	56.7%	61	48.0%	0.395	-
	Control	13	43.3%	66	52.0%		
**Total**		30	100.0%	127	100.0%		

HTN: arterial hypertension; T2DM: type 2 diabetes mellitus; HDL: high-density lipoprotein; *: statistically significant.

**Table A2:** Comparison of cardiometabolic comorbidities between lichen planus and psoriasis groups with odds ratios and 95% confidence intervals.

		HTN				Pearson Chi-Squared Test *p*-Value	OR 95% CI
		Present		Absent			
		N	%	N	%		
**Group**	Lichen planus	38	57.6%	40	44.0%	0.092	-
	Psoriasis	28	42.4%	51	56.0%		
**Total**		66	100.0%	91	100.0%		
		**T2DM**				**Pearson Chi-squared test** ***p*** **-value**	**OR** **95% CI**
		**present**		**absent**			
		**N**	**%**	**N**	**%**		
**Group**	Lichen planus	18	60.0%	60	47.2%	0.209	-
	Psoriasis	12	40.0%	67	52.8%		
**Total**		30	100.0%	127	100.0%		
		**Hypercholesterolemia**				**Pearson Chi-squared test** ***p*** **-value**	**OR** **95% CI**
		**present**		**absent**			
		**N**	**%**	**N**	**%**		
**Group**	Lichen planus	22	48.9%	56	50.0%	0.900	-
	Psoriasis	23	51.1%	56	50.0%		
**Total**		45	100.0%	112	100.0%		
		**Mixed dyslipidemia**				**Pearson Chi-squared test** ***p*** **-value**	**OR** **95% CI**
		**present**		**absent**			
		**N**	**%**	**N**	**%**		
**Group**	Lichen planus	12	75.0%	66	46.8%	0.033 *	3.409
	Psoriasis	4	25.0%	75	53.2%		1.049 ÷ 11.083
**Total**		16	100.0%	141	100.0%		
		**Low HDL**				**Pearson Chi-squared test** ***p*** **-value**	**OR** **95% CI**
		**present**		**absent**			
		**N**	**%**	**N**	**%**		
**Group**	Lichen planus	7	35.0%	71	51.8%	0.160	-
	Psoriasis	13	65.0%	66	48.2%		
**Total**		20	100.0%	137	100.0%		
		**Hypertriglyceridemia**				**Pearson Chi-squared test** ***p*** **-value**	**OR** **95% CI**
		**present**		**absent**			
		**N**	**%**	**N**	**%**		
**Group**	Lichen planus	3	37.5%	75	50.3%	0.719	-
	Psoriasis	5	62.5%	74	49.7%		
**Total**		8	100.0%	149	100.0%		
		**Abdominal obesity**				**Pearson Chi-squared test** ***p*** **-value**	**OR** **95% CI**
		**present**		**absent**			
		**N**	**%**	**N**	**%**		
**Group**	Lichen planus	17	32.7%	61	58.1%	0.003 **	0.350
	Psoriasis	35	67.3%	44	41.9%		0.174 ÷ 0.704
**Total**		52	100.0%	105	100.0%		

HTN: arterial hypertension; T2DM: type 2 diabetes mellitus; HDL: high-density lipoprotein; *: statistically significant; **: highly statistically significant.

**Table A3:** Odds ratios and *p*-values for cardiometabolic comorbidities in LP subgroups versus control and psoriasis groups.

		HTN				Pearson Chi-Squared Test *p*-Value	OR 95% CI
		Present		Absent			
		N	%	N	%		
**Group**	EOLP	18	40.9	11	17.2	0.006 **	3.336
	Control	26	59.1	53	82.8		1.377 ÷ 8.081
**Total**		44	100	64	100		
	EOLP	18	39.1	11	17.7	0.013 *	2.981
	Psoriasis	28	60.9	51	82.3		1.236 ÷ 7.189
**Total**		46	100	62	100		
**Group**	ROLP	29	52.7	25	32.1	0.017 *	2.365
	Control	26	47.3	53	67.9		1.161 ÷ 4.817
**Total**		55	100	78	100		
**Group**	ROLP	29	50.9	25	32.9	0.037 *	2.113
	Psoriasis	28	49.1	51	67.1		1.043 ÷ 4.281
**Total**		57	100	76	100		
**Group**	CLP	22	45.2	31	36.9	0.314	
	Control	26	54.2	53	63.1		
**Total**		48	100	84	100		
**Group**	CLP	22	44.0	31	37.8	0.481	
	Psoriasis	28	56.0	51	62.2		
**Total**		50	100	82	100		
**Group**	CMLP	13	33.3	15	22.1	0.202	
	Control	26	66.7	53	77.9		
**Total**		39	100	68	100		
**Group**	CMLP	13	31.7	15	22.7	0.304	
	Psoriasis	28	68.3	51	77.3		
**Total**		41	100	66	100		
		**T2DM**				**Pearson Chi-squared test** ***p*-value**	**OR** **95% CI**
		**Present**		**Absent**			
		**N**	**%**	**N**	**%**		
**Group**	EOLP	6	46.2	23	24.2	0.106	
	Control	7	53.8	72	75.8		
**Total**		13	100	95	100		
**Group**	EOLP	6	33.3	23	25.6	0.563	
	Psoriasis	12	66.7	67	74.4		
**Total**		18	100	90	100		
**Group**	ROLP	13	65.0	41	36.2	0.016 *	3.261
	Control	7	35.0	72	63.7		1.205 ÷ 8.827
**Total**		20	100	113	100		
**Group**	ROLP	13	52.0	41	38.0	0.198	
	Psoriasis	12	48.0	67	62.0		
**Total**		25	100	108	100		
**Group**	CLP	9	56.3	44	37.9	0.161	
	Control	7	43.8	72	62.1		
**Total**		16	100	116	100		
**Group**	CLP	9	42.9	44	39.6	0.783	
	Psoriasis	12	57.1	67	60.4		
**Total**		21	100	111	100		
**Group**	MCLP	3	30.0	25	25.8	0.719	
	Control	7	70.0	72	74.2		
**Total**		10	100	97	100		
**Group**	MCLP	3	20.0	25	24.3	0.516	
	Psoriasis	12	80.0	67	75.7		
**Total**		15	100	92	100		
		**Hypercholesterolemia**				**Pearson Chi-squared test** ***p*-value**	**OR** **95% CI**
		**Present**		**Absent**			
		**N**	**%**	**N**	**%**		
**Group**	EOLP	10	43.5	19	22.4	0.043 *	2.672
	Control	13	56.5	66	77.6		1.013 ÷ 7.046
**Total**		23	100	85	100		
**Group**	EOLP	10	30.3	19	25.7	0.383	
	Psoriasis	23	69.7	56	74.3		
**Total**		33	100	75	100		
**Group**	ROLP	18	58.1	36	35.3	0.024 *	2.538
	Control	13	41.9	66	64.7		1.117 ÷ 5.769
**Total**		31	100	102	100		
**Group**	ROLP	18	43.9	36	39.1	0.605	
	Psoriasis	23	56.1	56	60.9		
**Total**		41	100	92	100		
**Group**	CLP	16	55.2	37	35.9	0.062	
	Control	13	44.8	66	64.1		
**Total**		29	100	103	100		
**Group**	CLP	16	41.0	37	39.8	0.894	
	Psoriasis	23	59.0	56	60.2		
**Total**		39	100	93	100		
**Lot**	MCLP	10	43.5	18	21.4	0.033 *	2.821
	Control	13	56.5	66	78.6		1.064 ÷ 7.480
**Total**		23	100	84	100		
**Lot**	MCLP	10	30.3	18	24.3	0.516	
	Psoriasis	23	69.7	56	75.7		
**Total**		33	100	74	100		
		**Mixed dyslipidemia**				**Pearson Chi-squared test** ***p*-value**	**OR** **95% CI**
		**Present**		**Absent**			
		**N**	**%**	**N**	**%**		
**Group**	EOLP	3	25.0	26	27.1	0.878	
	Control	9	75.0	70	72.9		
**Total**		12	100	96	100		
**Group**	Group	3	42.9	26	25.7	0.383	
	Group	4	57.1	75	74.3		
**Total**		7	100	101	100		
**Group**	ROLP	4	30.8	50	41.7	0.447	
	Control	9	69.2	70	58.3		
**Total**		13	100	120	100		
**Group**	ROLP	4	50.0	50	40.0	0.715	
	Psoriasis	4	50.0	75	60.0		
**Total**		8	100	125	100		
**Group**	CLP	9	50.0	44	38.6	0.359	
	Control	9	50.0	70	61.4		
**Total**		18	100	114	100		
**Group**	CLP	9	23.5	44	37.0	0.024 *	3.835
	Psoriasis	4	76.5	75	63.0		1.115 ÷ 13.190
**Total**		13	100	119	100		
**Group**	MCLP	1	10.0	27	27.8	0.449	
	Control	9	90.0	70	72.2		
**Total**		10	100	97	100.0		
**Group**	MCLP	1	20.0	27	26.5	1.000	
	Psoriasis	4	80.0	75	73.5		
**Total**		5	100	102	100		
		**Low HDL**				**Pearson Chi-squared test** ***p*-value**	**OR** **95% CI**
		**Present**		**Absent**			
		**N**	**%**	**N**	**%**		
**Group**	EOLP	4	23.5	25	27.5	1.000	
	Control	13	76.5	66	72.5		
**Total**		17	100	91	100		
**Group**	EOLP	4	23.5	25	27.5	1.000	
	Psoriasis	13	76.5	66	72.5		
**Total**		17	100	91	100		
**Group**	ROLP	6	31.6	48	42.1	0.387	
	Control	13	68.4	66	57.9		
**Total**		19	100	114	100		
**Group**	ROLP	6	31.6	48	42.1	0.387	
	Psoriasis	13	68.4	66	57.9		
**Total**		19	100	114	100		
**Group**	CLP	4	23.5	49	42.6	0.134	
	Control	13	76.5	66	57.4		
**Total**		17	100	115	100		
**Group**	CLP	4	23.5	49	42.6	0.134	
	Psoriasis	13	76.5	66	57.4		
**Total**		17	100	115	100		
**Group**	MCLP	3	18.8	25	27.5	0.553	
	Control	13	81.3	66	72.5		
**Total**		16	100	91	100		
**Group**	MCLP	3	18.8	25	27.5	0.553	
	Psoriasis	13	81.3	66	72.5		
**Total**		16	100	91	100		
		**Hypertriglyceridemia**				**Pearson Chi-squared test** ***p*-value**	**OR** **95% CI**
		**Present**		**Absent**			
		**N**	**%**	**N**	**%**		
**Group**	EOLP	0	0	29	27.4	1.000	
	Control	2	100	77	72.6		
**Total**		2	100	106	100		
**Group**	EOLP	0	0	24	28.2	0.321	
	Psoriasis	5	100	44	71.8		
**Total**		5	100	68	100		
**Group**	ROLP	2	20.0	52	40.3	1.000	
	Control	2	50.0	77	59.7		
**Total**		4	100	129	100		
**Group**	ROLP	2	28.6	52	41.3	0.700	
	Psoriasis	5	71.4	74	58.7		
**Total**		7	100	126	100		
**Group**	CLP	2	50.0	51	39.8	1.000	
	Control	2	50.0	77	60.2		
**Total**		4	100	128	100		
**Group**	CLP	2	28.6	51	40.8	0.701	
	Psoriasis	5	71.4	74	59.2		
**Total**		7	100	125	100		
**Group**	MCLP	1	33.3	27	26.0	1.000	
	Control	2	66.7	77	74.0		
**Total**		3	100	104	100		
**Group**	MCLP	1	16.7	27	26.7	1.00	
	Psoriasis	5	83.3	74	73.3		
**Total**		6	100	101	100		
		**Abdominal obesity**				**Pearson Chi-squared test** ***p*-value**	**OR** **95% CI**
		**Present**		**Absent**			
		**N**	**%**	**N**	**%**		
**Group**	EOLP	5	27.8	11	17.7	1.000	
	Control	13	72.2	51	82.3		
**Total**		18	100	62	100		
**Group**	EOLP	5	12.5	24	35.3	0.010 *	0.262
	Psoriasis	35	87.5	44	64.7		0.091 ÷ 0.757
**Total**		40	100	68	100		
**Group**	ROLP	12	48.0	42	38.9	0.403	
	Control	13	52.0	66	61.1		
**Total**		25	100	108	100		
**Group**	ROLP	12	25.5	42	2	0.009 **	0.359
	Psoriasis	35	74.5	44	51.		0.165 ÷ 0.784
**Total**		47	100	86	100		
**Group**	CLP	14	51.9	39	37.1	0.164	
	Control	13	48.1	66	62.9		
**Total**		27	100	105	100		
**Group**	CLP	14	28.6	39	47.0	0.037	0.451
	Psoriasis	35	71.4	44	53.0		0.212 ÷ 0.960
**Total**		49	100	83	100		
**Group**	MCLP	9	40.9	19	22.4	0.078	
	Control	13	59.1	66	77.6		
**Total**		22	100	85	100		
**Group**	MCLP	9	20.5	19	30.2	0.261	
	Psoriasis	35	79.5	44	69.8		
**Total**		44	100	63	100		

HTN: arterial hypertension; T2DM: type 2 diabetes mellitus; HDL: high-density lipoprotein; *: statistically significant; **: highly statistically significant.

**Table A4:** Comparative analysis of cardiometabolic comorbidities in ROLP versus controls and psoriasis.

		HTN				Pearson Chi-Squared Test *p*-Value	OR 95% CI
		Present		Absent			
		N	%	N	%		
**Group**	ROLP	29	52.7%	25	32.1%	0.017 *	2.365
	Control	26	47.3%	53	67.9%		1.161 ÷ 4.817
**Total**		55	100.0%	78	100.0%		
		**T2DM**				**Pearson Chi-squared test** ***p*** **-value**	**OR** **95% CI**
		**present**		**absent**			
		**N**	**%**	**N**	**%**		
**Group**	ROLP	13	65.0%	41	36.3%	0.016 *	3.261
	Control	7	35.0%	72	63.7%		1.205 ÷ 8.827
**Total**		20	100.0%	113	100.0%		
		**Hypercholesterolemia**				**Pearson Chi-squared test** ***p*** **-value**	**OR** **95% CI**
		**present**		**absent**			
		**N**	**%**	**N**	**%**		
**Group**	ROLP	18	58.1%	36	35.3%	0.024 *	2.538
	Control	13	41.9%	66	64.7%		1.117 ÷ 5.769
**Total**		31	100.0%	102	100.0%		
		**Mixed dyslipidemia**				**Pearson Chi-squared test** ***p*** **-value**	**OR** **95% CI**
		**present**		**absent**			
		**N**	**%**	**N**	**%**		
**Group**	ROLP	4	30.8%	50	41.7%	0.447	-
	Control	9	69.2%	70	58.3%		
**Total**		13	100.0%	120	100.0%		
		**Low HDL**				**Pearson Chi-squared test** ***p*** **-value**	**OR** **95% CI**
		**present**		**absent**			
		**N**	**%**	**N**	**%**		
**Group**	ROLP	6	31.6%	48	42.1%	0.387	-
	Control	13	68.4%	66	57.9%		
**Total**		19	100.0%	114	100.0%		
		**Hypertriglyceridemia**				**Pearson Chi-squared test** ***p*** **-value**	**OR** **95% CI**
		**present**		**absent**			
		**N**	**%**	**N**	**%**		
**Group**	ROLP	2	50.0%	52	40.3%	1.000	-
	Control	2	50.0%	77	59.7%		
**Total**		4	100.0%	129	100.0%		
		**Abdominal obesity**				**Pearson Chi-squared test** ***p*** **-value**	**OR** **95% CI**
		**present**		**absent**			
		**N**	**%**	**N**	**%**		
**Group**	ROLP	12	48.0%	42	38.9%	0.403	-
	Control	13	52.0%	66	61.1%		
**Total**		25	100.0%	108	100.0%		
		**HTN**				**Pearson Chi-squared test** ***p*** **-value**	**OR** **95% CI**
		**present**		**absent**			
		**N**	**%**	**N**	**%**		
**Group**	ROLP	29	50.9%	25	32.9%	0.037 *	2.113
	Psoriasis	28	49.1%	51	67.1%		1.043 ÷ 4.281
**Total**		57	100.0%	76	100.0%		
		**T2DM**				**Pearson Chi-squared test** ***p*** **-value**	**OR** **95% CI**
		**present**		**absent**			
		**N**	**%**	**N**	**%**		
**Group**	ROLP	13	52.0%	41	38.0%	0.198	-
	Psoriasis	12	48.0%	67	62.0%		
**Total**		25	100.0%	108	100.0%		
		**Hypercholesterolemia**				**Pearson Chi-squared test** ***p*** **-value**	**OR** **95% CI**
		**present**		**absent**			
		**N**	**%**	**N**	**%**		
**Group**	ROLP	18	43.9%	36	39.1%	0.605	-
	Psoriasis	23	56.1%	56	60.9%		
**Total**		41	100.0%	92	100.0%		
		**Mixed dyslipidemia**				**Pearson Chi-squared test** ***p*** **-value**	**OR** **95% CI**
		**present**		**absent**			
		**N**	**%**	**N**	**%**		
**Group**	ROLP	4	50.0%	50	40.0%	0.715	-
	Psoriasis	4	50.0%	75	60.0%		
**Total**		8	100.0%	125	100.0%		
		**Low HDL**				**Pearson Chi-squared test** ***p*** **-value**	**OR** **95% CI**
		**present**		**absent**			
		**N**	**%**	**N**	**%**		
**Group**	ROLP	6	31.6%	48	42.1%	0.387	-
	Psoriasis	13	68.4%	66	57.9%		
**Total**		19	100.0%	114	100.0%		
		**Hypertriglyceridemia**				**Pearson Chi-squared test** ***p*** **-value**	**OR** **95% CI**
		**present**		**absent**			
		**N**	**%**	**N**	**%**		
**Group**	ROLP	2	28.6%	52	41.3%	0.700	-
	Psoriasis	5	71.4%	74	58.7%		
**Total**		7	100.0%	126	100.0%		
		**Abdominal obesity**				**Pearson Chi-squared test** ***p*** **-value**	**OR** **95% CI**
		**present**		**absent**			
		**N**	**%**	**N**	**%**		
**Group**	ROLP	12	25.5%	42	48.8%	0.009 **	0.359
	Psoriasis	35	74.5%	44	51.2%		0.165 ÷ 0.784
**Total**		47	100.0%	86	100.0%		

ROLP: reticular oral lichen planus; HTN: hypertension; T2DM: type 2 diabetes mellitus; HDL: high-density lipoprotein; *: statistically significant; **: highly statistically significant.

## References

[1] OECD & European Commission (2024). Health at a Glance: Europe 2024: State of Health in the EU Cycle.

[2] Orlando Molon B., Viola A., Alaibac M., Angioni R., Piaserico S. (2022). Psoriasis and Cardiovascular Diseases: An Immune-Mediated Cross Talk?. Front. Immunol..

[3] Garshick M.S., Ward N.L., Krueger J.G., Berger J.S. (2021). Cardiovascular Risk in Patients with Psoriasis: JACC Review Topic of the Week. J. Am. Coll. Cardiol..

[4] Anyfanti P., Margouta A., Goulas K., Gavriilaki M., Lazaridou E., Patsatsi A., Gkaliagkousi E. (2022). Endothelial Dysfunction in Psoriasis: An Updated Review. Front. Med..

[5] Lockshin B., Balagula Y., Merola J. (2018). Interleukin-17, Inflammation, and Cardiovascular Risk in Patients with Psoriasis. J. Am. Acad. Dermatol..

[6] Tylutka A., Morawin B., Walas Ł., Michałek M., Gwara A., Zembron-Lacny A. (2023). Assessment of metabolic syndrome predictors in relation to inflammation and visceral fat tissue in older adults. Sci. Rep..

[7] National Cholesterol Education Program (NCEP) Expert Panel (2002). Third Report of the National Cholesterol Education Program (NCEP) Expert Panel on Detection, Evaluation, and Treatment of High Blood Cholesterol in Adults (Adult Treatment Panel III) final report. Circulation.

[8] International Diabetes Federation (IDF) (2006). The IDF Consensus Worldwide Definition of the Metabolic Syndrome.

[9] Pirowska M., Obtułowicz A., Lipko-Godlewska S., Goździalska A., Podolec K., Wojas-Pelc A. (2018). The level of proinflammatory cytokines: Interleukins 12, 23, 17 and tumor necrosis factor α in patients with metabolic syndrome accompanying severe psoriasis and psoriatic arthritis. Postepy. Dermatol. Alergol..

[10] Barros G., Duran P., Vera I., Bermúdez V. (2022). Exploring the Links between Obesity and Psoriasis: A Comprehensive Review. Int. J. Mol. Sci..

[11] Ikeda K., Morizane S., Akagi T., Hiramatsu-Asano S., Tachibana K., Yahagi A., Iseki M., Kaneto H., Wada J., Ishihara K. (2022). Obesity and Dyslipidemia Synergistically Exacerbate Psoriatic Skin Inflammation. Int. J. Mol. Sci..

[12] Kumar S.A., Krishnam Raju P.V., Gopal K.V.T., Rao T.N. (2019). Comorbidities in Lichen Planus: A Case–control Study in Indian Patients. Indian Dermatol. Online J..

[13] Gonzalez Navarro B., Egido Moreno S., Omaña Cepeda C., Estrugo Devesa A., Jane Salas E., Lopez Lopez J. (2024). Relationship between Oral Lichen Planus and Cardiovascular Disease of Atherosclerotic Origin: Systematic Review and Meta-Analysis. J. Clin. Med..

[14] Toader M.P., Taranu T., Constantin M.M., Olinici D., Mocanu M., Costan V.V., Toader S. (2021). High serum level of interleukin-6 is linked with dyslipidemia in oral lichen planus. Exp. Ther. Med..

[15] Mathur M., Thakur N., Jaiswal S., Das G., Shah S., Maharjan S., Paudel S., Shrestha A., Upadhyay H.P. (2023). Metabolic syndrome in patients with lichen planus: A case-control study. Skin Health Dis..

[16] Daye M., Temiz S.A., Isık B. (2021). The relationship between lichen planus and metabolic syndrome. J. Cosmet. Dermatol..

[17] Tashiro T., Sawada Y. (2022). Psoriasis and Systemic Inflammatory Disorders. Int. J. Mol. Sci..

[18] Xia J., Ding L., Liu G. (2025). Metabolic syndrome and dermatological diseases: Association and treatment. Nutr. Metab..

[19] Pradhan A.D., Manson J.E., Rifai N., Buring J.E., Ridker P.M. (2001). C-reactive protein, interleukin 6, and risk of developing type 2 diabetes mellitus. J. Am. Med. Assoc..

[20] Sutherland J., McKinley B., Eckel R. (2004). The Metabolic Syndrome and Inflammation. Metab. Syndr. Relat. Disord..

[21] Vičić M., Hlača N., Kaštelan M., Brajac I., Sotošek V., Prpić Massari L. (2023). Comprehensive Insight into Lichen Planus Immunopathogenesis. Int. J. Mol. Sci..

[22] Hashba H., Bifi J., Thyvalappil A., Sridharan R., Sreenivasan A., Mathew P. (2018). Prevalence of Metabolic Syndrome in Patients with Lichen Planus: A Cross-sectional Study from a Tertiary Care Center. Indian Dermatol. Online J..

[23] Danielsen K., Wilsgaard T., Olsen A.O., Eggen A., Olsen K., Cassano P., Furberg A. (2015). Elevated odds of metabolic syndrome in psoriasis: A population-based study of age and sex differences. Br. J. Dermatol..

[24] Angeli F., Bucciarelli V., Moscucci F., Sciomer S., Ricci F., Coppi F., Bergamaschi L., Armillotta M., Alvarez M.C., Renda G. Gender and Sex-related differences in Type 2 Myocardial Infarction: The undervalued side of a neglected disease. Trends Cardiovasc Med.

[25] Rather P.A., Tilwani M.R., Khan Z.A. (2022). Association between lichen planus and dyslipidemia: An experience from North India. EUREKA Health Sci..

[26] Kuntoji V., Kudligi C., Bhagwat P.V., Manasa D.R., Sharma A., Andanappanavar V., Asati D.P., Dhayaneethi E. (2016). Dyslipidemia and metabolic syndrome in patients with lichen planus: A case-control study. J. Pak. Assoc. Dermatol..

[27] Singla R., Ashwini P.K., Jayadev B. (2019). Lichen Planus and Metabolic Syndrome: Is There a Relation?. Indian Dermatol. Online J..

[28] De Porras-Carrique T., Ramos-García P., González-Moles M.Á. (2024). Hypertension in oral lichen planus: A systematic review and meta-analysis. Oral Dis..

[29] Rodríguez-Fonseca L., Llorente-Pendás S., García-Pola M. (2023). Risk of Prediabetes and Diabetes in Oral Lichen Planus: A Case–Control Study according to Current Diagnostic Criteria. Diagnostics.

[30] Hu Y., Zhu Y., Lian N., Chen M., Bartke A., Yuan R. (2019). Metabolic Syndrome and Skin Diseases. Front. Endocrinol..

[31] Nowowiejska J., Baran A., Flisiak I. (2022). Lipid Aberrations in Lichen Planus. Metabolites..

[32] European Academy of Dermatology and Venereology Task Force (2013). Clinical practice guideline for an integrated approach to comorbidity in patients with psoriasis. J. Eur. Acad. Dermatol. Venereol..

[33] Elmets C.A., Leonardi C.I., Davis D.M.R., Gelfand J.M., Lichten J., Mehta N.N., Armstrong A.W., Connor C., Cordoro K.M., Elewski B.E. (2019). Joint AAD–NPF guidelines of care for the management and treatment of psoriasis with awareness and attention to comorbidities. J. Am. Acad. Dermatol..

